# Genetic Basis of Breast and Ovarian Cancer: Approaches and Lessons Learnt from Three Decades of Inherited Predisposition Testing

**DOI:** 10.3390/genes15020219

**Published:** 2024-02-08

**Authors:** Valeria Barili, Enrico Ambrosini, Beatrice Bortesi, Roberta Minari, Erika De Sensi, Ilenia Rita Cannizzaro, Antonietta Taiani, Maria Michiara, Angelica Sikokis, Daniela Boggiani, Chiara Tommasi, Olga Serra, Francesco Bonatti, Alessia Adorni, Anita Luberto, Patrizia Caggiati, Davide Martorana, Vera Uliana, Antonio Percesepe, Antonino Musolino, Benedetta Pellegrino

**Affiliations:** 1 Department of Medicine and Surgery, University of Parma, 43126 Parma, Italy; 2 Medical Genetics, University Hospital of Parma, 43126 Parma, Italy; 3 Medical Oncology Unit, University Hospital of Parma, 43126 Parma, Italy; 4 Breast Unit, University Hospital of Parma, 43126 Parma, Italy

**Keywords:** hereditary breast and ovarian cancer (HBOC) syndrome, *BRCA1* and *BRCA2*, homologous recombination deficiency (HRD), poly (ADP-ribose) polymerase (PARP) inhibitors

## Abstract

Germline variants occurring in *BRCA1* and *BRCA2* give rise to hereditary breast and ovarian cancer (HBOC) syndrome, predisposing to breast, ovarian, fallopian tube, and peritoneal cancers marked by elevated incidences of genomic aberrations that correspond to poor prognoses. These genes are in fact involved in genetic integrity, particularly in the process of homologous recombination (HR) DNA repair, a high-fidelity repair system for mending DNA double-strand breaks. In addition to its implication in HBOC pathogenesis, the impairment of HR has become a prime target for therapeutic intervention utilizing poly (ADP-ribose) polymerase (PARP) inhibitors. In the present review, we introduce the molecular roles of HR orchestrated by BRCA1 and BRCA2 within the framework of sensitivity to PARP inhibitors. We examine the genetic architecture underneath breast and ovarian cancer ranging from high- and mid- to low-penetrant predisposing genes and taking into account both germline and somatic variations. Finally, we consider higher levels of complexity of the genomic landscape such as polygenic risk scores and other approaches aiming to optimize therapeutic and preventive strategies for breast and ovarian cancer.

## 1. Introduction

Breast cancer is the most prevalent type of cancer in women worldwide and the existence of a familial predisposition has been known for at least 150 years [1]. The co-segregation of familial ovarian and breast cancer was described in the second part of the 20th century. In 1994, through extensive research on families with a history of early-onset breast and/or ovarian cancer, the discovery of the connection between BRCA1 and BRCA2 genes and the development of cancer risk was made [2,3]. Shortly after, *BRCA1/2* screening became available for clinical use with an immediate effect on patient care [4].

In the last few years, the list of genes involved in cancer predisposition has increased, reflecting various consequences from the prognostic, preventive, and therapeutic point of view. These genes can be ranked in two categories according to their contribution to cancer risk: high penetrance, where the risk is more than fourfold higher than the one observed in the overall population, and intermediate penetrance, with a 2–4 times higher risk [5]. Among the high-penetrance hereditary cancer syndromes, *BRCA1*- and *BRCA2*-Associated Hereditary Breast and Ovarian Cancer (HBOC) still stands out as the most prevalent [6].

The acronym HBOC is used to define a genetic condition mainly characterized by an enhanced susceptibility to both female and male breast and ovarian tumors, such as fallopian tube and peritoneal malignancies. HBOC is historically linked to germline pathogenic variants in *BRCA1* and *BRCA2*, which have vital functions in fixing double-stranded DNA error. Additionally, genetic variants associated with HBOC have been described to enhance risks for prostate cancer and pancreatic cancer.

The approximate lifetime probability of breast tumor development in the overall population is around 12%. In two extensive studies conducted prospectively within cancer genetics services, it was determined that by the age of 70, individuals carrying *BRCA1* pathogenic variants faced a breast cancer risk ranging from 60% to 66%, while those with *BRCA2* pathogenic variants exhibited a risk between 55% and 61%. The estimated incidence of ovarian cancer in the overall population is approximately 1–2%. Ovarian cancer risk was calculated to be between 41% and 58% for *BRCA1* carriers and between 15% and 16.5% for *BRCA2* carriers at age 70 [7,8].

The prevalence of HBOC is estimated to be around 1:400 individuals [9]. However, individuals with Ashkenazi Jewish ancestry have a significantly higher prevalence (1:40), attributable to three Jewish founder pathogenic changes, that eventually led to evaluation of a potential population screening [10].

Traditionally, HBOC risk is based on assessing the family history, particularly the presence of several individuals who have been diagnosed with breast and/or ovarian tumor occurring at young ages. The variability in the response to standard breast cancer treatment, together with faster and cheaper sequencing technologies, has prompted a directional shift, with the implementation of multigene panels and the use of genetic analysis also for therapeutic purposes.

This review covers the possible role of pathogenic variants in breast and ovarian cancer screening, prevention, and monitoring with a possible improvement in more-focused therapeutic interventions.

## 2. High-Penetrance Genes: *BRCA1* and *BRCA2*

### 2.1. Pathogenic Mechanism

DNA repair encompasses various mechanisms, such as DNA single-strand break (dsDNA) repair or base mismatch repair (MMR). Notably, *BRCA1* and *BRCA2* stand as tumor-suppressor genes essential to the preservation of genomic integrity through the mending of dsDNA lesions employing homologous recombination (HR). In addition, they guide centrosome dynamics and the distribution of chromosome, cytokinesis, and genome stabilization throughout cell cycle progression [11].

A hormone-dependent carcinogenic environment that could potentially compromise genome stability through the disruption of BRCA functions has been hypothesized. This disruption might consequently lead to the expedited induction of pro-survival cues, thereby promoting the transformation of breast gland cells into transformed phenotypes [12]. Due to the correlation between the impairment of BRCA activity and defects in HR, the conceptual framework of “BRCAness” was introduced in 2004 [13]. This term denotes a phenocopy of *BRCA1* or *BRCA2* variants, a situation in which an HRR defect exists in a tumor in the absence of germline *BRCA1* or *BRCA2* changes. Several conceptually distinct approaches to detect non-*BRCA1/2* gene variants could also result in the same consequences caused by those detected in *BRCA1/2*. Therefore, the concept of BRCAness could serve as a valuable therapeutic biomarker.

Moreover, it has been shown that tumors associated with *BRCA1* pathogenic variants often exhibit a molecular profile reminiscent of the Triple-Negative Breast Cancer (TNBC) subtype, while tumors linked to *BRCA2* variants tend to resemble the Luminal B subtype or, less commonly, the Luminal A subtype [14].

### 2.2. Types of Variants

Currently, the ClinVar database [15] has identified around 4300 different germline variants in *BRCA1* and 5200 in *BRCA2*, classified as pathogenic or likely pathogenic (as considered in December 2023). About 80% of these variants are categorized as truncating modifications, which result in the formation of premature stop codons, including frameshift and nonsense changes. It must be noted that the removal of the terminal amino acids cannot necessarily impact on protein function, as stated by Nepomuceno et al. through functional evaluations of the impact of frameshift variants in the C-term of BRCA1, emphasizing the need for a comprehensive assessment of individual variants beyond their primary sequence alterations [16].

Missense variants constitute approximately 10% of the identified variants [17]. Their interpretation can be troublesome, as many of them are still categorized as variants of uncertain significance (VUSs) in line with the American College of Medical Genetics (ACMG) guidelines [18]. Pathogenic missense variants are typically localized within functionally crucial domains, such as the Really Interesting New Gene (RING) finger domain and the BRCA1 C terminus (BRCT) domains of BRCA1, as well as the regions spanning the oligosaccharide-binding folds (OBs) and helical domains of BRCA2. Intriguingly, by investigating the number of pathogenic missense variants in those domains with respect to the whole protein, *BRCA1* includes approximately half of the variants with RING and BRCT domains (76/162), in contrast to *BRCA2* which shows around 70% of missense changes (56/78) within the helicase and OB domains.

Moreover, amino acid modifications at distinct positions, e.g., first methionine or the regions near the exon–intron junctions, are usually considered pathogenic according to the ACMG classification.

Notably, about 10% of the variants are attributed to aberrant copy number variations (CNVs), ascertained through deletion or duplication analyses [19]. Moreover, a mechanism termed “secondary epimutation”, due to the presence of the BRCA1 deep intronic variant c.-107A > T, may lead to the methylation of the promoter and to a reduced expression of the gene [20].

### 2.3. Genotype–Phenotype Correlations

*BRCA1* is positioned at locus 17q21.31 and comprises a total of 24 exons, encoding a sequence of 1863 amino acids (MIM #113705). The initiation of the exonic portion occurs with exon 2, wherein the primary transcript omits exon 4. Specific regions known as the breast cancer cluster regions (BCCRs) within *BRCA1* are localized at coordinates c.179–505, c.4328–4945, and c.5261–5563. Likewise, the ovarian cancer cluster region (OCCR) resides at coordinates c.1380–4062 [21].

The genomic location of *BRCA2* is on chromosome 13 q13.1 and it encompasses 27 exons, encoding a peptide sequence spanning 3418 amino acids. The protein-coding region starts within exon 2. The breast cancer cluster regions (BCCRs) within *BRCA2* are identified at positions c.1–596, c.772–1806, and c.7394–8904. In a parallel manner, the ovarian cancer cluster regions (OCCRs) are positioned within the coordinates c.2831–6401 and c.6645–7471 [21]. Variants within these regions appear to trigger nonsense-mediated decay (NMD), resulting in the loss of *BRCA2* expression and a markedly elevated susceptibility of ovarian cancer. In fact, the most prominent disparity observed between the risks observed in carriers with diverse ethnic backgrounds and those identified in individuals of Ashkenazi Jewish descent pertains to the heightened ovarian cancer risk associated with *BRCA2* pathogenic variants in AJ populations. This discrepancy can be due to the data concerning the *BRCA2* founder variant in AJ, which reflects the susceptibility linked to the 6174delT variant, which is situated within the OCCR of the BRCA2 gene [7,22]. Interestingly, *BRCA2* showed a strong risk association with pathogenic variants and prostate and pancreatic cancer. Specifically, *BRCA2* germline variants within the region c.6373–c.6492 exhibited a relative risk of 3.7 as compared with molecular changes outside this region [23], and pathogenic changes after the 3′ of c.7914 (prostate cancer cluster region or PrCCR) were significantly associated with an augmented risk of prostate cancer [24]. Additionally, a pancreatic cancer cluster region (PcCCR) was identified as associated with pancreatic cancer (between c.3515 and c.6787), a region which overlaps with *BRCA2* OCCR but not BCCR and PrCCR, suggesting a possible common oncogenic mechanism between ovarian and pancreatic tumors [25]. On the other hand, specific variants seem to be associated with a better prognosis. For example, pathogenic variants within *BRCA1* exon 11 have the potential to generate a partial BRCA1 protein due to the presence of known exon 11 splice variants, though the entire protein is subjected to degradation via NMD. Experimental evidence from murine embryos harboring the exon 11-deleted isoform indicates extended survival compared to embryos lacking BRCA1 entirely, suggesting that BRCA1 proteins lacking exon 11 may retain some degree of functional activity [26].

Another example is the pathogenic variant p.Lys3326Ter in *BRCA2*, correlated with a reduced possibility of breast and ovarian tumor when compared to other pathogenic *BRCA2* variants [27]. However, the study reported an odds ratio (OR) of 1.28 for breast cancer and an OR of 1.26 for invasive ovarian cancer. Hence, in the absence of supplementary data, the utilization of genotype location and its correlation with the phenotype of cancer risk for the purpose of individual risk evaluation and management might be considered premature.

Considering that the genetic tests for *BRCA1* and *BRCA2* have more than doubled in the last decade, the importance of accessible and high-quality data becomes crucial.

Thus, the most extensive initiative named “BRCA Exchange project” (www.brcaexchange.org) started to create a comprehensive and global source of information on BRCA genetic variants. The project is dedicated to compiling and consolidating data related to *BRCA1* and *BRCA2* variants, defined for a high-penetrance phenotype, from diverse sources and populations worldwide. The goal is to establish a centralized and expansive repository of knowledge on *BRCA* genetic variations that can be accessed and utilized by researchers, clinicians, and professionals globally [28]

## 3. Other High-Penetrance Genes

The discovery of additional genes associated with breast and/or ovarian cancer risk has prompted their involvement in tumor-associated gene panels, and it has fostered the development of evolving frameworks aimed at optimizing the management of carriers. Across several studies involving high-risk HBOC patients, where individuals were not pre-selected for *BRCA1/2* mutation status, the frequency of variants in non-*BRCA1/2* genes ranges from 3.5% to 10.9% [29]. In other breast cancer patient cohorts with no selection bias, the rate of detection of variants in *BRCA1/2* was approximately 4–5%, while the cumulative variant detection rate achieved through multigene panel analysis ranged from 6% to 9%, representing a 1.4- to 2-fold augmentation. Likewise, among ovarian cancer patients free from selection biases, the rate of detection of variants in *BRCA1*/*BRCA2* was roughly 20%, whereas the rate for MGP analysis ranged from 26% to 31%, corresponding to a 1.5-fold elevation [30,31].

Recent comprehensive studies have highlighted that pathogenic variants (PVs) across eight distinct genes, such as *ATM*, *BRCA1*, *BRCA2*, *CHEK2*, *PALB2 (FANCN)*, *RAD51C*, and *RAD51D*, exhibit a substantial correlation with breast cancer risk (Table 1). Notably, PVs within the *BRCA1*, *BRCA2*, and *PALB2* genes are associated with a markedly elevated BC risk, with odds ratios spanning from 5.0 to 10.6. Conversely, PVs within the *ATM* and *CHEK2* genes confer a moderately elevated BC risk, with odds ratios ranging from 2.1 to 2.5 [32,33]. It is pertinent to note, however, that variants in *ATM* and *CHEK2* are comparatively more prevalent within the general population [34].

Ultimately, *TP53* and *CDH1* can be included among high-penetrance genes. Germline variants in *BRCA1* and *TP53* are primarily connected with invasive ductal carcinoma, whereas *BRCA2* germline changes are linked to both ductal and lobular tumors. In contrast, variants in *CDH1* are uniquely associated with lobular breast cancer, particularly the invasive type [35].

### 3.1. CDH1

Germline variants in CDH1 (E-cadherin) tumor suppressor protein are correlated with hereditary diffuse gastric cancer (HDGC) and a lifetime risk of 40–50% of developing lobular breast cancer [36,37]. Annual mammography and bilateral breast MRI with contrast are recommended beginning at age 30. Risk-reducing mastectomy depends on family history [38].

Inactivating germline variants in *CDH1* underlie this high-penetrance cancer syndrome. These germline changes are identified in the 40% of families that meet the HDGC clinical criteria. The cumulative incidence of DGC by the age of 80 among *CDH1* variants carriers is calculated as around 70% for males and 56% for females, while the likelihood of women developing LBC stands at 42%. Other clinical manifestations, including colorectal cancer (CRC), appendiceal signet ring cell carcinomas, and cleft lip/palate, have also been observed in HDGC families.

In the clinical context of HDGC, *CDH1* variants are uniformly scattered across the gene, and no discernible genotype–phenotype correlation has been identified [39]. The predominance of documented germline modifications has been described to result in truncated proteins or in the absence of mRNA transcription, thereby facilitating a straightforward assessment of their pathogenicity. Missense variants constitute 16% of the described changes thus far: the clinical and functional implications of missense variants are debated among geneticists, in most instances, since a full-length protein is retained, and normal E-cadherin protein expression is typically maintained.

In 2013, a study involving 165 unrelated cases was conducted [37]. The selection criteria were based on personal and familial histories of gastric or breast cancers, and the findings revealed that 18 subjects (11%) were carriers of *CDH1* variants, encompassing 18 distinct variants. Among these carriers, three women had personal histories of bilateral LBC diagnosed before the age of 50. None of these cases exhibited a family history of diffuse gastric cancer among first- and second-degree family members, and therefore did not satisfy the HDGC rules set forth in that period. Subsequently, two of these women were diagnosed with DGC, while the third woman underwent upper gastrointestinal endoscopy with several bioptic sampling. Significantly, in recent years, a growing body of research has emerged, documenting instances of early-onset LBC cases in individuals carrying *CDH1* germline variants, even in the absence of any personal or family history of DGC. This highlights *CDH1* as a newly identified susceptibility gene for LBC. Furthermore, the concept of hereditary lobular breast cancer (HLBC) has been suggested as a distinct clinical entity linked to *CDH1* germline modifications. According to the consensus guidelines established for HDGC, women who meet specific criteria, including those diagnosed with bilateral LBC, regardless of family cases of LBC prior to the age of 50, or women diagnosed with unilateral LBC with other cases of LBC in the family, characterized below 45 years, meet the necessary criteria for *CDH1* genetic testing [40].

### 3.2. PALB2

The PALB2 gene participates in the HR process as a binding partner of *BRCA2*. Biallelic modifications in *PALB2,* also known as *FANCN*, led to a distinct type of Fanconi anemia, while monoallelic *PALB2* variants are linked to breast cancer and ovarian cancer with a 41–60% and 3–5% absolute risk, respectively [41,42]. The prevalence of these pathogenic variants ranges from 0.4% to 3.9%. *PALB2* pathogenic variants are conducive to a 35% lifetime risk of developing breast tumor by the age of 70.

The NCCN guidelines recommend annual mammography and MRI with contrast starting at the age of 30 for women with the *PALB2* pathogenetic variant [42]. Risk-reducing mastectomy (RRM) is another option to consider, whereas data about Risk-reducing Salpingo-oophorectomy (RRSO) are lacking, and the presence of other family cases should be considered [43].

Further increased risks have been observed for pancreatic cancer, ranging from 2% to 3%, and male breast cancer with a 1% risk [44].

A systematic review [45] revealed that the majority of the cataloged predicted pathogenic PALB2 variants, specifically 911 cases (92.5%), were documented in breast cancer patients, while 49 cases (5.0%) were observed in patients with ovarian tumors, and 24 cases (2.4%) were recorded in patients with pancreatic cancer. Notably, most frequent pathogenic *PALB2* variants were c.509_510delGA, c.3113G > A, c.1592delT, c.172_175delTTGT, and c.1240C > T, collectively representing 57.3% of all cases. The “hotspots” exonic regions with the highest variant frequencies were in exons 2 (representing 6.7%), 1 (reflecting 6.3%), and 3 (5.8%).

### 3.3. PTEN

Tumor-suppressor gene PTEN pathogenic germline variants cause Cowden syndrome/PTEN Hamartoma Tumor Syndrome (PHTS), an autosomal dominant disease featured by multiple hamartomas and an increased risk of developing certain cancers in the thyroid, skin, endometrium, and breast [46]. The PHTS-estimated prevalence is currently at 1:200,000, with many cases which remain undetected [47]. The clinical presentation of PHTS is highly heterogeneous and encompasses an elevated risk for various cancer types, as well as macrocephaly, developmental delays, cutaneous manifestations, thyroid abnormalities, vascular malformations, and benign tumors [48].

Current population-based estimates for PHTS indicate that females exhibit a cancer risk of 85–90%, and males face a risk of 54–66% by the age of 60. Specifically, elevated risks have been noted for breast cancer (67–78%), endometrial cancer (19–28%), thyroid cancer (6–38%), colorectal cancer (9–20%), renal cancer (2–11%), and melanomas (0–6%) [49].

NCCN guidelines recommend 6–12-month breast inspections starting at the age of 25, and annual mammography and breast MRI screening with contrast starting at 30–35 years of age [42,43,46]. Thyroid cancer surveillance commences in childhood, at age 18, or upon PHTS diagnosis. Guidance for endometrial, colorectal, renal cancer, and melanoma surveillance varies, ranging from no recommended surveillance to annual monitoring [50].

Concerning the genotype–phenotype correlation, truncating variants exhibit a 2- to 3-fold higher risk of breast cancer (BC) when compared to missense variants [51]. Furthermore, variants located in the phosphatase domain are associated with an additional approximate 2-fold increased BC risk compared to those situated in the C2 domain. Although the precise underlying mechanisms driving these genotype-specific BC risks still need clarification, these statistically significant findings underscore the necessity of assessing genotype-related surveillance strategies. Particularly, it is imperative to consider the potential reduction in breast cancer monitoring for patients harboring missense variants in the C2 domain, as these changes are more frequently ascertained in individuals with (mild) developmental delay than in patients with high cancer risk [52].

### 3.4. TP53

The tumor suppressor gene TP53 is situated on chromosome 17p13, and its protein product, p53, assumes significant roles in the regulation of both the cell cycle and apoptosis. Li–Fraumeni syndrome (LFS) is a rare autosomal dominant disorder due to germline *TP53* pathogenic variants and characterized by high cancer predisposition for a wide range of tumors, including brain cancer, sarcomas, acute leukemia, and early-onset breast cancer [41]. The occurrence of breast tumor in *TP53*-carrying women is remarkably high with a cumulative incidence at 60 years of 85%; screening suggests clinical breast surveillance every 6–12 months starting from 20 years of age, annual breast MRI with contrast from 20 to 75 years, and annual mammography from 30 to 75 years [42,46]. A clear association between pathogenetic variants in *TP53* and ovarian cancer has not been established [42].

Soft tissue sarcoma, breast cancer, adrenocortical tumors, and specific types of brain tumors have been designated as the “core” cancers within the LFS spectrum [53]. The NCI Li–Fraumeni Syndrome Study, encompassing 5286 individuals, demonstrated cumulative incidence rates by the age of 70 in women, indicating 54% for breast cancer, 15% for soft tissue sarcoma, 6% for brain cancer, and 5% for osteosarcoma [54]. In a comprehensive case–control analysis involving 56,480 breast tumors, *TP53* pathogenic variants exhibited a significant association with HER2-positive disease, irrespective of estrogen receptor status [55]. Collectively, these results imply that HER2 amplification may coincide with germline *TP53* changes. This correlation demands additional exploration, as individuals with this dual characteristic may potentially derive benefits from chemoprevention strategies incorporating HER2-targeted agents.

Irrespective of familial medical histories, the prevalence of disease-causing germline *TP53* changes stands between 3.8% and 7.7% in females diagnosed with breast carcinoma before the age of 31 years [56]. The occurrence of presentations without a familial cancer history can be attributed to two main factors: the contribution of de novo variants to the h*TP53*-related cancer syndrome, estimated to range from 7 to 20%, and the lacking in the penetrance of pathogenetic *TP53* changes [57].

The absence of detectable *TP53* variants on blood DNA analysis also through high-depth NGS does not ensure the absence of somatic alterations. In this context, systematically, a comprehensive screening should encompass the analysis of tumor tissue. Interestingly, blood detection of a low number of TP53 variant sequences may be due to circulating tumor DNA (e.g., metastatic tumors) or clonal hematopoiesis, which relies on somatic *TP53* alterations which benefit hematopoietic stem cells, highlighting the complexity of mosaic variants in *TP53* [58]. The detection of a *TP53* variant in the bloodstream of a patient afflicted with metastatic high-grade serous ovarian carcinoma is more likely indicative of circulating tumor DNA. This presumption is based on the exceedingly high prevalence of somatic *TP53* alterations in such malignancies, which surpasses 95%. Secondly, clonal hematopoiesis presents the second potential pitfall, encompassing the emergence of somatic *TP53* alterations that confer a proliferative advantage within hematopoietic stem cells. Initially, clonal hematopoiesis was documented primarily in patients aged 70 and older, but it has been detected in individuals as young as 30 years of age. The prevalence of clonal hematopoiesis escalates with age and is influenced by factors such as tobacco use, as well as exposure to chemotherapy or radiotherapy [59]. Therefore, when a *TP53* variant is identified within a minor fraction of sequencing reads derived from blood, it is imperative to consider the clinical presentation and relevant aspects of the medical history, including prior treatments and the presence of metastases, and to attest the presence of the variant within the tissue of origin of the tumor.

The penetrance of germline variants in *TP53* which cause the disease exhibits variability, often determined by the type of molecular change. Dominant-negative missense *TP53* changes are typically detected in families characterized by pediatric tumors and commonly display high penetrance. In contrast, truncating/frameshift variants are primarily found in families predominantly affected by adult tumors and show reduced disease penetrance. A notable case of a low-penetrance yet still pathogenic variant is the non-dominant negative missense p.Arg337His variant, which occurs in 0.3% of the Southern Brazilian population and is correlated with a founder genetic effect [60].

The observed phenotypic variability in disease expression within the same family strongly suggests the presence of genetic modifying factors, even environmental ones. Identifying these factors currently stands as a top priority in this genetic field. Germline *TP53* variants can render p53 more permissive to oncogenic stress, highlighting the interplay between genetic and environmental factors in determining the ultimate clinical outcome.

## 4. Intermediate Penetrance Genes

### 4.1. ATM

The ataxia-telangiectasia-mutated (ATM) gene specifies a kinase engaged in the DNA double-strand break repair mechanism. *ATM* pathogenic variants, such as homozygous or compound heterozygous changes, cause ataxia telangiectasia, a syndromic disease characterized by progressive cerebellar ataxia, oculomotor apraxia, immunodeficiency, and enhanced cancer predisposition [61]. The severe and recessive syndromic form is rare; however, variant carrier frequency is not. *ATM* pathogenic variants are observed in heterozygosity in approximately 1–2% of Caucasian adults [62].

Women carrying a heterozygous pathogenetic variant in *ATM* have a 20–40% lifetime risk to develop breast cancer; thus, annual mammography is recommended from 40 years [42,46]. Data regarding RRM are not sufficient yet, so this option is considered based on family history [42]. Germline pathogenetic variants confer a 2-fold to 3-fold increase in the risk of ovarian cancer compared to the general population [63], but there are no available data to recommend RRSO [42].

Heterozygosity for loss-of-function variants in *ATM* has been correlated with an increased susceptibility to prostate, pancreatic, gastric, colorectal, and skin cancers too [64,65,66].

Recent outcomes from an international and a US-based investigation have provided evidence that both truncating and missense molecular changes of *ATM* associate with an augmented risk of ER-positive BC compared to ER-negative BC [32,33].

The available findings do not display a significantly elevated risk of contralateral breast cancer among carriers of *ATM* pathogenic variants in comparison to non-carriers. Currently, a few studies indicate a limited increase in risk among *ATM* PV carriers [67]. Further data are required to accurately evaluate the CBC risk in people with ATM variants.

Some genotype–phenotype correlations have been demonstrated. The *ATM* missense variant c.7271T > G, initially associated with a milder phenotype of ataxia-telangiectasia (A-T), has been found to carry a substantial breast cancer risk comparable to that of *BRCA2* [68,69].

The interplay between radiation exposure and breast cancer risk is intricate for patients harboring *ATM* PVs. Ataxia-telangiectasia-affected patients exhibit a heightened sensitivity to ionizing radiation. However, available data generally do not contraindicate for patients with heterozygous *ATM* PVs and radiation therapy. The Women’s Environmental Cancer and Radiation Epidemiology (WECARE) study examines the interaction between radiation exposure, genetic predisposition, and breast cancer, specifically radiation-induced CBC. Women carrying a common *ATM* variant may experience a protective effect, reducing CBC risk. Conversely, women with rare, likely deleterious *ATM* missense variants face an elevated dose-dependent CBC risk compared to *ATM* PV carriers who did not undergo RT [67,70]. The efficacy of PARPi in breast cancer (BC) patients with *ATM* pathogenic variants (PVs) is currently under examination within the metastatic setting. The TBCRC048 phase II study revealed no notable activity among *ATM* carriers; however, the trial cohort included only a limited number of *ATM* carriers [71]. Olaparib obtained approval for metastatic prostate cancer patients carrying pathogenic variants in DNA repair genes, including *ATM*, primarily based on the De Bono et al. study. It is worth noting that specific activity in *ATM* carriers was not studied in that trial [72].

### 4.2. BARD1

The BARD1 gene (BRCA1-associated RING domain 1) possesses both structural and functional resemblances with the BRCA1 protein, playing pivotal roles in DNA repair and apoptosis functions [73]. Germline variants in *BARD1* exhibited a higher risk of 2- to 4-fold to develop breast cancer as compared to population-based risk [74], but there is insufficient evidence for RRM [42]. A cancer predisposition due to *BARD1* pathogenic variants is in fact relatively rare, occurring in fewer than 1 in 500 BC patients [75].

Shimelis et al. conducted comprehensive tests on triple-negative BC, demonstrating a significant association between germline PVs in *BARD1* and a notably elevated risk of TNBC. Additionally, the risk for BC over a lifetime exceeded 20% [76].

Among a cohort of 222 patients diagnosed with aggressive neuroblastoma, two germline *BARD1*-truncating variants were identified [77]. This discovery prompts an inquiry into the potential involvement of *BARD1* variants in the context of high-risk neuroblastoma. Investigations have been conducted to explore the plausible role of BARD1 in ovarian cancer (OC); however, the current body of evidence is insufficient to establish a definitive association with increased OC risk [78].

### 4.3. BRIP1

The BRCA1-interacting protein 1 (BRIP1) gene specifies for a helicase protein, which interacts with the C-terminal domain of BRCA1 and plays a role in the BRCA1-dependent DNA repair process, particularly concerning interstrand cross-link (ICL) damage repair.

At present, *BRIP1* germline modifications are not clearly associated with an increased risk for breast cancer; conversely, there is a strong association with ovarian cancer with an absolute lifetime risk up to 15% and risk-reducing surgery is recommended at 45–50 years of age [42]. *BRIP1* has been observed as the third-most commonly associated gene with ovarian cancer susceptibility, with nearly 0.9% to 2.5% of all ovarian cancer patients carrying a variant.

Germline *BRIP1* variants, either in homozygosity or compound heterozygosity, are correlated with a subtype of Fanconi anemia (FANCJ) [79].

Its role as a BC-predisposing gene was first reported in 2006 through a gene-based method for families with hereditary BC that could not be attributed to pathogenic variants in *BRCA1* and *BRCA2* [80]. However, subsequent independent studies have not consistently shown an association between PVs in *BRIP1* and BC, leaving the role of *BRIP1* in BC risk uncertain [81].

These *BRIP1* variants are found in fewer than 1–5% of familial and sporadic OC or BC cases, a significantly lower prevalence compared to pathogenic *BRCA1* and *BRCA2* variants [82].

While pathogenic variants in *BRIP1* have been characterized, genetic testing often reveals missense variants with uncertain effects on molecular function and cancer risk. Germline analysis from 2160 early-onset breast tumor patients and 1199 ovarian cancer patients displayed that nearly 2% of these individuals carry very rare missense variants in *BRIP1* (allele frequency < 0.0001), with a frequency that is three times higher than that of all rare *BRIP1* missense variants found in over 60,000 subjects from general individuals (*p* < 0.0001). Moreover, Moyer et al. functionally characterized 20 missense variants, within the helicase domain, using the CRISPR/Cas9 strategy and rescue assays, evaluating the impaired protein’s stability and affecting the repair of ICL damage. Notably, 75% of the studied variants were found to render the protein hypomorphic or nonfunctional. In a clinical gene-panel-tested cohort comprising over 117,000 BC- and OC-affected patients, the combined odds ratio associated with carriers of *BRIP1* hypomorphic or nonfunctional missense variants compared to the general population was 2.30 (1.60–3.30; *p* < 0.0001), highlighting the importance of functional testing to assess the impact of such variants [83].

### 4.4. CHEK2

The CHEK2 (Checkpoint Kinase 2) gene encodes for a tumor-suppressor protein involved in DNA repair, cell cycle arrest, and apoptosis as a response to DNA damage. It is classified as a moderate-penetrance breast cancer risk gene. Heterozygosity for *CHEK2* pathogenic variants is observed in approximately 1% of European descendants. Different alterations in *CHEK2* have been documented, including 1100delC (the most extensively analyzed), I157T, R117G, I160 M, and G167R. By studying Dutch and Finnish people, *CHEK2* 1100delC is prevalent, whereas p.S428F is more common in Ashkenazi Jews and less commonly found in women from Asia [33]. Notably, the most prevalent *CHEK2* truncating molecular changes (1100delC and del5395) are associated with over a 2-fold increased BC risk with a lifetime risk ranging from 28 to 37%, depending on the family history [84]. Women carrying these variants should undergo annual mammography and MRI starting from age 40, and RRM may be considered based on family history [42,46]. The association with *CHEK2* variants and ovarian cancer is not clear, so, at the moment, there are no specific protocols or recommendations for RRSO [43]. Biallelic 1100delC was linked to a more than two-fold augmented risk of BC compared to heterozygotes, suggesting the need for intensive breast cancer surveillance [85].

The missense change I157T, predominantly in Finland and Poland, correlates with a 1.4-fold BC risk [86].

Recent case–control studies showed that *CHEK2* PVs related to protein-truncating changes are highly associated with ER-positive BC compared to ER-negative ones [32]. Additionally, truncating *CHEK2* variant carriers exhibited a positive familial history and higher incidence of bilateral BC [87]. In another study from England, a significant correlation between the CHEK2*1100delC PVs and pure ductal carcinoma in situ (DCIS) was reported, whereas no such association was observed with missense variants [88].

Muranen et al. conducted an analysis to assess the potential combined effect of the *CHEK2* 1100delC variant and 77 common germline changes related to the polygenic risk score (PRS) on breast cancer risk. Their study demonstrated that the PRS could effectively establish a high-risk subgroup of *CHEK2* 1100delC PV carriers who may have an advantage from clinical interventions [89].

Besides BC, *CHEK2* pathogenic variants have been linked to various tumor types, including colorectal cancer, prostate cancer [90], renal cell carcinoma [91], thyroid cancer [92], testicular germ cell tumors [93], and male breast cancer [94]. The potential association for melanoma and *CHEK2* PVs is currently under examination [95]. The missense change I157T has been linked to ovarian tumors of benign, borderline, and low-grade malignancy [96].

### 4.5. RAD51C and RAD51D

The RAD51 gene paralogs are involved in DSB repair, HR, and NHEJ [46]. Pathogenetic variants in *RAD51C* and *RAD51D* are linked to a lifetime risk up to 20–40% for BC and management consists of annual mammography and contrast MRI starting at age 40. The risk of ovarian cancer for *RAD51C* and *RAD51D* pathogenic variants carriers is up to 10–20% and RRSO is recommended at 45–50 years or earlier based on other family cases [42].

Within a Finnish study, a recurring PV in *RAD51D* (c.576+1G>A) was identified among patients affected with both BC and OC [97].

Notably, Shimelis et al. highlighted a novel association between TNBC and *RAD51D*. Within their study, the authors recognized five TNBC predisposing genes, such as *RAD51D*, characterized by a cumulative lifetime risk exceeding 20% for breast cancer [76].

Another study identified the c.270_271dupTA as a recurring variant and revealed that *RAD51D* pathogenic variant carriers with TNBC exhibited characteristics such as positive axillary lymph nodes and high-grade tumors. Furthermore, these carriers displayed an aggressive type and early onset BC, with an average age comparable to that of patients with *BRCA* pathogenic variants [98].

## 5. Variants of Uncertain Significance

A primary limitation of multigene panel implementation is the risk of finding a higher number of variants of uncertain significance (VUSs). These are usually missense variants, uncommon in the population (not defined as polymorphisms) and lacking clear evidence of altering protein function. Due to their uncertain clinical significance, VUSs are not “actionable”, and clinical management typically relies on family history. However, identifying VUSs can lead to significant anxiety and raise the risk of clinical misinformation, including the improper execution of management approaches exclusively for individuals with known pathogenic variants.

As the number of genes included in panel testing increases, the number of VUSs also rises [99]. Moreover, the number of VUSs can be influenced by the calling and variant algorithms employed by the testing laboratory. Due to the variability in variant annotations used by different panel studies, it is challenging to rigorously calculate an overall VUS rate for multigene panel testing. Some earlier estimates have suggested that up to 88.4% of patients may have at least one VUS, but further research is required to refine this understanding [100]. Importantly, the American College of Medical Genetics and Genomics (ACMG) and the Association for Molecular Pathology (AMP) in 2015 realized a guideline for the interpretation of germline variants [18] relevant for genes, inheritance patterns, and diseases. The 28 defined criteria categorize the type of variant evidence toward a benign or pathogenic effect and the level of strength such as strong, moderate, or supporting, based mainly on population frequency data, variant type, location in the protein, segregation, functional, and computational information [18]. In this context, a VUS becomes etiopathogenic in relation to the disease with a probabilistic range which varies from 0.10 to 0.90. To determine the effective association, the ACMG/AMP settled strong evidence for functional validation of the protein activity associated with that variant, determined by the PS3 and BS3 criteria. However, discordances between laboratories in the application of this criteria were evident depending on the tested assays. Thus, in 2020, The Clinical Genome Resource (ClinGen) Sequence Variant Interpretation (SVI) Working Group highlighted specific definitions for the use of PS3/BS3 by calculating a predictive value (OddsPath) on validated controls to provide robust evidence on the various functional assays [101]. Not of lesser importance, a pathogenicity parameter to apply for germline variant evaluation relies on in silico functional prediction, based mainly on the knowledge obtained by computer simulation/model analysis. The algorithms evaluate sequence phylogenetic conservation in both evolutionary and interspecific contexts, biochemical/structural variables, splice-site, and unsupervised machine learning. Some of the most important available tools are SIFT, which analyzes all the possible amino acid changes to compromise protein function [102]; CADD (https://cadd.gs.washington.edu/) [103]; PolyPhen-2 (http://genetics.bwh.harvard.edu/pph2/index.shtml) [104]; Mutation Taster (https://spliceailookup.broadinstitute.org/) [105]; AlphaFold2 [106,107]; Alphamissense [108]; REVEL [109]; and SpliceAI (https://spliceailookup.broadinstitute.org/) [105].

In recent decades, the advent of high-throughput multiplexed assays for variant effect (MAVEs), which collected a significant number of germline variant genotypes, helped in directly associating the molecular alteration with its effect through functional assays [110]. The MAVE project aims to assess and characterize the functional effects of genetic variants in the BRCA genes. The initiative employs a multiplexed assay approach, involving a series of simultaneous functional tests, to analyze and better understand the impacts of genetic variants. This involves using a series of tests that evaluate different aspects of the gene’s function, with the goal of gaining a more comprehensive understanding of the effects of genetic variants. The outcomes of MAVE have significantly contributed to the classification of genetic variants in BRCA genes, providing crucial information for a more accurate assessment of the associated risk with these variants. MAVEs are a set of techniques encompassing deep mutational scanning experiments on proteins and massively parallel reporter assays on gene regulatory sequences. MAVE experiments have collected sequence–function associations with a base-pair resolution through deep mutational scans and for regulatory elements via massively parallel reporter assays.

With the advent of CRISPR/Cas9 technology and other gene-editing tools, novel methods are emerging that can directly introduce variants into endogenous gene loci. This idea was first shown by Findlay et al. (2014), who mutagenized specific codons in a single exon of *BRCA1,* measured transcript abundance by targeted RNA sequencing, or analyzed reduced cellular fitness by targeted DNA sequencing [111]. In 2018, Findlay et al. applied genome editing to assess the functionality of every potential SNV in crucial regions of *BRCA1*, irrespective of prior human observations. They use the saturation genome editing of *BRCA1* by coupling multiplex-homology-directed repair with CRISPR/Cas9 RNA-guided cleavage using a composite library of donor templates to show the saturation editing of genomic regions.

The functional effects of nearly 4000 SNVs were found to be in perfect concordance with established assessments of pathogenicity. Furthermore, they identified over 400 non-functional missense SNVs, as well as approximately 300 SNVs that disrupt expression [112].

Using a similar approach, Sahu et al. classified 599 *BRCA2* variants, including 93 SNVs across 11 codons, 28 of which are documented in ClinVar. Additionally, they functionally classified 252 SNVs from exon 13 into 188 functional variants and 4 intermediate and 60 non-functional variants [113]. Recently, a functional characterization, based on saturation genome editing (SGE), allowed 97% of all putative SNVs in the *BRCA2* DNA-binding domain (DBD) to be evaluated for pathogenic missense variants. Thanks to this study, the number of SNVs in *BRCA2* DBD, previously classified by ClinVar as Pathogenic/Likely Pathogenic (n = 417) or Benign/Likely Benign (n = 993), increases from 1410 to 5818 SNVs classified [114]. Moreover, Fayer et al. discovered that approximately half of the *BRCA1* VUSs tested through SGE could be clinically reclassified as “likely pathogenic” or “likely benign” [115].

Similar to *BRCA1* and *BRCA2*, numerous other variants can be deciphered in tumor suppressor genes (e.g., PALB2, RAD51D, RAD51C). Indeed, ongoing efforts are being made to perform SGE on many other genes, and this information is accessible through the online platform MaveDB, a public repository for datasets derived from MAVEs (https://www.mavedb.org/#/) [116].

Currently, additional functional assays have been conducted to assess the impact of certain VUSs in these genes. For instance, Boonen et al. employed a combination of functional tests in *PALB2* and *CHEK2*. Their evaluation demonstrated that having VUSs in the Coiled-Coil domain of *PALB2* can disrupt the binding with *BRCA1*, while VUSs in the VD40 region interfere with the protein’s inherent stability [117,118].

Concerning *CHEK2*, the authors employed a system based on mouse embryonic stem cells to quantitatively determine the functional impact of 50 missense VUSs. Assessing the activity of human *CHEK2* in phosphorylating one of its key targets, it was discovered that 31 missense VUSs in *CHEK2* compromise protein function to a similar extent as truncating variants, while 9 missense VUSs yielded intermediate results. Furthermore, this study reveals that most VUSs impair the function of the CHK2 kinase by causing protein instability or compromising activation through (auto)phosphorylation. Quantitative results demonstrated that the CHK2 kinase dysfunction severity correlates with an increased risk of breast cancer [118].

What emerges from these studies is that SGE is a viable strategy for functionally classifying thousands of variants in a clinically relevant gene. The use of a scoring system to assess hundreds of observed variants can provide immediate functional evaluations for newly observed ones.

The issue of VUSs is pertinent for the racial and ethnic minority community, in which a higher proportion of VUSs are determined in multigene panels compared to non-Caucasians [99]. This higher VUS rate is partly attributed to the higher representation of Caucasians in reference databases, which results from a well-documented referral bias, leading to less testing of minority populations. Additionally, populations with higher genetic variability, such as Africans and African Americans, can contribute to an increased rate of VUSs due to their lack of a historical population bottleneck [119]. These challenges in variant classification point out the value of executing genetic testing in collaboration with experienced genetics professionals.

To improve consistency, numerous private laboratories contribute to peer-reviewed databases like ClinVar [15] and collaborate in shared genomics data projects, facilitated by consortia like ClinGen [120], the Global Alliance for Genomics and Health (http://genomicsandhealth.org), and Atlas of Variants Alliance (https://www.varianteffect.org/) [121]. Moreover, the Evidence-based Network for the Interpretation of Germline Mutant Alleles (ENIGMA) transnational alliance was instituted to address the *BRCA1-2* VUS issue (https://enigmaconsortium.org/). ENIGMA stands as a research-driven, globally collaborative entity recognized as an authoritative expert panel by ClinVar. Its principal objective is to enhance research endeavors and methodologies for the classification of variants in *BRCA1*, *BRCA2*, and additional genes associated with breast and ovarian cancer susceptibility. The consortium has devised criteria for the classification of variants that encompass both statistics-based quantitative and rules-based qualitative methodologies, tailored to evaluate the clinical evaluation of variants in *BRCA1* and *BRCA2*. The quantitative variants grouping is grounded in multifactorial probability models encompassing parameters such as population data, clinical assessments, and predictions derived from bioinformatics. Notably, the consortium advances the practice of sharing data from expansive-scale projects complemented by variant annotations [122,123].

The study of Fayer et al. explores the outcomes of a comprehensive multiplexed functional data curation initiative, a rigorous assessment for helping in resolving VUSs for clinical value. The evaluation encompasses genetic variants in *BRCA1*, *TP53*, and *PTEN* genes, employing a variety of assays for assessment. In the context of *BRCA1*, the functional data effectively differentiated between pathogenic and benign variants, providing robust evidence in favor of both classifications. *TP53* variants underwent evaluation using a classifier trained on four distinct assays, yielding accurate predictions and supporting evidence codes that contributed to the overall strength of interpretation. Despite limitations in sensitivity, the *PTEN* data played a role in reclassifying certain variants. Thus, this study extends its focus to variant reinterpretation, demonstrating the substantial impact of incorporating multiplexed functional data into the classification process. This impact is particularly pronounced in cases where conflicting evidence exists, emphasizing the value of such data in refining variant interpretations. The research underscores the potential utility of integrating multiplexed functional data with a machine-learning approach into clinical assessments, offering a nuanced and comprehensive understanding of genetic variant effects for improved clinical decision making [115].

Lastly, to fully validate VUSs in BRCA genes, functional assays should be employed by detecting homologous recombination activity in patient-derived primary fibroblasts and/or by *in vitro* overexpression of the variants. Particularly, treatment with X-rays, UV radiation, PARP inhibitors, mitomycin C, cisplatin, topoisomerase inhibitors, and alkylating agents induces DNA recombination, and the various events may be evaluated, such as phosphorylated histone H2AX and RAD51 foci, and the detection of DNA repair synthesis with BrdU incorporation. Nonetheless, ambiguity persists regarding the extent to which the functional assessment of HR activity can serve as a predictor for the susceptibility of tumor cells harboring *BRCA1* or *BRCA2* variants to the effects of PARP inhibitors and DNA-damaging agents.

The complexities surrounding VUSs have emerged as a significant argument against the implementation of genetic screening. The incidence of VUSs tends to escalate with the inclusion of a greater number of genes in a testing panel. Furthermore, they are more prevalent in populations that have been less extensively studied and in genes that have only recently come under investigation. We contend that this concern can be effectively addressed through a governance decision not to report VUSs in the context of screening. This guideline is acceptable because the primary purpose of screening is not to identify every individual at risk. In fact, the nonreporting of VUSs is already a standard practice in preconception carrier analysis, the reporting of other incidental findings in genomic tests, and the disclosure of data in clinical biobanks. This approach may limit sensitivity, leading to a specific degree of incorrect negatives, and it offers two pivotal advantages: (a) it enhances specificity, thereby reducing incorrect positives, and (b) in the case of BRCA screening, it may empower policymakers to increase testing efforts and improve the currently inadequate proportion of carrier identification [124].

### Functional Assays of Specific HRR Genes (Table 2)

To discriminate between pathogenic variants and VUSs in HRR genes, several functional assays have been developed that evaluate how the variant impacts on the gene activity.

*BRCA1 functional assays* [125]

Different functional assays have been used to assess the impact of genetic variants of uncertain significance (VUSs) in the BRCA1 gene. These techniques aim to classify VUSs based on their impact on protein conformation or function.

- Ubiquitin ligase activity and protein interaction: this combines ubiquitin ligase activity and yeast two-hybrid assays to assess the variant impact on the BRCA1 RING domain in mediating interactions.
- Transcription Activation (TA) assay: a quantitative assay that measures the impact of variants on transactivation by the acidic C-terminal region of *BRCA1* on reporter genes.

Other functional assays:

- Protease sensitivity assay: this can be used to detect VUSs that affect protein folding.
- Phosphopeptide binding assays: this can be used to study the interaction of BRCA1 BRCT domains with phosphorylated peptides.
- Small-Colony Phenotype (SCP) assay: this reveals how *BRCA1* expression impacts on yeast growth.
- Yeast Localization Phenotype (YLP) assay: this can be used to investigate the cellular localization of BRCA1 in yeast.
- ESC-based functional assay: this can be used to study the impact of VUSs by mouse embryonic stem cells.
- Restoration of radiation resistance: this can be used to investigate if *BRCA1* variants are able to restore radiation resistance.
- Homology-Directed Recombination (HDR) assay: this can be used to evaluate how VUSs impact on the correct functionality of the Homologous Recombination Repair (HRR) pathway.
- Centrosome amplification: this can be used to study how VUSs impact on centrosome amplification.
- Yeast recombination assay: by studying the yeast HRR pathway, this can be used to evaluate the effect of *BRCA1* missense VUSs.
- Subcellular localization assay: this can be used to observe how BRCA1 subcellular localization varies under the influence of VUS.

*BRCA2 functional assays* [126]

Several functional assays are available to analyze the effect of VUSs on the BRCA2 protein:

- Homology-Directed Recombination (HDR) assay: see previous paragraph.
- HRR assay in human cells: this can be used to evaluate the effect of transient overexpression of *BRCA2* VUSs on a recombination reporter substrate in HeLa G1 cells. As certain pathogenic variants exhibited effects similar to non-pathogenic ones, its specificity is uncertain.
- Yeast recombination assay: human full-length *BRCA2* is expressed in the yeast strain to measure HRR. HRR is increased by neutral variants, while it is decreased/is stable by pathogenic variants.
- Centrosome-amplification assay: as pathogenic *BRCA2* mutations induce increased centrosomes, while neutral variants and WT do not, this exhibits high specificity and reasonable sensitivity.
- Mitomycin C (MMC) survival assay: this can be used to evaluate the activity of MMC on cells harboring *BRCA2* VUSs (cell lines with pathogenic mutations are more sensitive to MMC).
- Embryonic stem cell (ESC)-based functional assay: this can be used to study the ability of human *BRCA2* VUSs to rescue ES cell viability.
- Syngeneic human cancer *BRCA2* knockout cell line model (SyVal model): this can be used to evaluate RAD51 foci formation and sensitivity to DNA-damaging agents introducing *BRCA2* VUSs into a p53-deficient human epithelial colorectal cancer cell line.
- Nuclear localization assay: as pathogenic variants exhibit cytoplasmic localization, while non-pathogenic variants remain nuclear, this can be used to observe the subcellular localization of GFP-tagged *BRCA2* variants to discriminate between VUSs and pathogenic mutations.
- BRCA2 protein–protein-interaction-based assays: this can be used to study how *BRCA2* VUSs modify the interactions with other proteins, such as PALB2.
- Analysis of variants that affect RNA splicing.
- Phenotype in heterozygous carriers: cells from *BRCA2* heterozygous variant carriers and healthy controls seem to behave differently upon DNA damage. Available data are still not robust to routinely use this assay to validate *BRCA2* VUSs.

*CHK2 functional assays*

VUSs in CHEK2 gene have been extensively studied in order to discriminate between pathogenic and non-pathogenic variants, supporting their clinical interpretation and clarifying their role in cancer predisposition. Here is a list of the main functional assays employed:

- *In vitro* kinase assays;
- Yeast strains expressing human *CHEK2;*
- Knockout of breast cell lines for *CHEK2*.

Using these tools, researchers have been able to classify almost 179 *CHEK2* VUSs as pathogenic or not. Nonetheless, a mechanistic follow-up is needed to confirm the functional evaluation [127].

*TP53 functional assays*

The following assays are currently used to validate *TP53* variants:

- Apoptotic pathway activation upon DNA damage: this evaluates the impact on the function of *TP53* variants by verifying the capacity of the protein in activating the apoptotic pathway upon DNA damage. The cell lines used to test the apoptotic response are peripheral blood lymphocytes administered with ionizing radiation.
- FASAY (Functional Assay for the Separation of Alleles in Yeast): this involves testing the ability of TP53 proteins to transactivate the ADE2 gene in yeast, providing a complementary approach to the apoptotic assay [128].

**Table 2:** Functional assays for the classification of variants of uncertain significance.

Assay	Description	Main Findings
***BRCA1* Functional Assays**		
Ubiquitin Ligase Activity and Protein Interaction	This combines ubiquitin ligase activity and yeast two-hybrid assays to assess variant impact on BRCA1 RING domain in mediating interactions.	VUSs influence on protein conformation and interactions.
Transcription Activation (TA) Assay	This is a quantitative assay that measures the impact of variants on transactivation by the acidic C-terminal region of *BRCA1* on the reporter gene.	VUSs affect *BRCA1* transcription activation.
Other Functional Assays	Protease sensitivity, phosphopeptide binding, SCP, YLP, ESC-based, restoration of radiation resistance, HDR, centrosome amplification, yeast recombination, subcellular localization.	VUSs impact on protein structure, cell cycle, and response to treatment.
***BRCA2* Functional Assays**		
Homology-directed repair (HDR) assay	This can be used to evaluate how VUSs impact on the correct functionality of Homologous Recombination Repair (HRR) pathway.	VUSs impact on HRR pathway.
Homologous recombination assay in human cells	This can be used to evaluate the effect of the transient overexpression of *BRCA2* VUS on a recombination reporter substrate in HeLa G1 cells.	VUSs affect intra-chromosomal recombination.
Yeast recombination assay	Human full-length *BRCA2* is expressed in the yeast strain to measure HRR.	HRR is increased by neutral variants, while it is decreased/is stable by pathogenic variants.
Centrosome-amplification assay	Pathogenic *BRCA2* mutations induce increased centrosomes, while neutral variants and WT do not.	It exhibits high specificity and reasonable sensitivity.
Mitomycin C (MMC) survival assay	This can be used to evaluate the activity of MMC on cells harboring *BRCA2* VUSs.	Cell lines with pathogenic mutations are more sensitive to MMC.
Embryonic stem cell (ESC)-based functional assay	This can be used to study the ability of human *BRCA2* VUSs to rescue ES cell viability.	High specificity.
Syngeneic human cancer *BRCA2* knockout cell line model	This can be used to evaluate RAD51 foci formation and sensitivity to DNA-damaging agents, introducing *BRCA2* VUSs into a *TP53*-deficient human epithelial colorectal cancer cell line.	VUSs impact on DNA damage response.
Nuclear localization assay	This can be used to observe the subcellular localization of GFP-tagged *BRCA2* variants.	Pathogenic variants exhibit cytoplasmic localization, while non-pathogenic variants remain nuclear.
BRCA2 protein–protein-interaction-based assays	This can be used to study how *BRCA2* VUSs modify the interactions with other proteins.	The readout is the interaction with other proteins, such as PALB2.
Analysis of variants that affect RNA splicing	This can be used to investigate the presence of aberrant RNA splicing.	VUSs may influence RNA splicing.
Phenotype in heterozygous carriers	Cells from *BRCA2* heterozygous mutation carriers and healthy controls seem to behave differently upon DNA damage.	Available data are still not robust to routinely use this assay to validate BRCA2 VUSs.
**CHEK2 Functional Assays**		
Various *in vitro* kinase assays, budding yeast strains, and mammalian cell lines	These can be used to evaluate how VUSs impact on protein structure and activity upon DNA damage.	Using these tools, researchers have been able to classify almost 179 *CHEK2* VUSs as pathogenic or not. Nonetheless, a mechanistic follow-up is needed to confirm the functional evaluation.
***TP53* Functional Assays**		
Apoptotic assay	This can be used to evaluate the impact on the function of *TP53* variants by verifying the capacity of the protein to activate the apoptotic pathway upon DNA damage.	The cell lines used to test the apoptotic response are peripheral blood lymphocytes administered with ionizing radiation.
FASAY (Functional Assay for the Separation of Alleles in Yeast)	This involves testing the ability of TP53 proteins to transactivate the *ADE2* gene in yeast.	This provides a complementary approach to the apoptotic assay.

## 6. Mainstreaming or Direct Genetic Testing

Two alternative models that may be relevant for unaffected subjects are mainstreaming and direct genetic testing. Mainstreaming involves enlisting healthcare professionals who are not specialized in genetics to initiate genetic testing, typically with the support of genetics specialists [129]. In accordance with the model, patients are conveyed to genetic counseling only after the completion of gene analysis, and specifically in the case of a positive or undetermined result. Mainstreaming has primarily been explored and applied within the oncology domain, specifically with oncologists directly referring OC patients for genetic analysis. Research on mainstreaming in OC patients has revealed significantly higher referral rates and uptake (ranging from 89% to 100%) compared to traditional GC (15% to 31%). Patient waiting times have been notably reduced too [130,131]. In this framework, it is intended that all carriers receive post-test GC from a specialist in genetics.

In theory, mainstreaming may be employed for unaffected subjects, where general practitioners, gynecologists, or breast surgeons may provide genetic analysis throughout surveillance or periodic consultations. Nevertheless, mainstreaming heavily relies on healthcare providers who are not specialized in genetics and has not been extended to the vast majority of non-cancer specialists. For instance, while gynecological and oncological surgeons in UK have embraced mainstreaming, mammary surgeons expressed concerns about their expertise in delivering GC and supporting patients in making testing decisions, along with reservations about the time commitment involved [132].

The prospect of providing genetic counseling as part of regular primary care has also been examined [133]. Primary-care providers have identified several barriers, primarily rooted in their limited knowledge and abilities to provide patient counseling regarding genetic risks and the proper stewardship. Related problems, inclusive of those related to ethical, social, and legal issues, have been posed particularly concerning the offering of cancer genetics care [134]. Moreover, an improvement in providers’ knowledge and confidence following educational interventions [135] or employing appropriate electronic instruments has been shown in different studies [136]. Nonetheless, mainstreaming to non-genetics specialists necessitates retraining to attain the requisite expertise. It would entail a substantial transfer of responsibilities from genetic counselors to other clinical professionals, necessitating a resolution of logistical challenges, particularly those related to staff resources and time allocation.

## 7. Polygenic Risk Score

Mendelian inheritance with higher or lower penetrance, however, accounts for only a small share of the whole breast cancer burden, leaving the majority of women, even with a positive family history for the disease, without the possibility of a specific risk prediction. However, over the past 10 years, significant growth has been made in determining germline variants or single-nucleotide polymorphisms (SNPs), which can be associated with the risk of developing breast cancer and can be used for the risk prediction. Over the last decade, genome-wide association studies (GWASs) have demonstrated that common single-nucleotide polymorphisms (SNPs) are involved in determining the susceptibility to common complex disorders, uncovering variants, which occur in disease-affected vs. unaffected controls at a significantly higher frequency (typically with *p*-values lower than 5 × 10^−7^). In a polygenic condition, if considered individually, most of these SNPs have a moderate effect on disease association and are not valuable for estimating disease risk; on the contrary, when combined together, they show differences in their frequencies in affected and non-affected breast cancer patients [137]. Fundamental to unmasking the relationship between the individual genetic variants and the disease development is understanding the disease genetic architecture, i.e., in the case of breast and ovarian cancers, the number and frequency of genetic variants possibly associated with the disease risk and the relative weight of each of them. A source of confusion on genetic architecture relies on the categorization of a disease as monogenic as opposed to polygenic, meaning that the occurrence of the disease depends on a single- or many-gene changes. From a genetic point of view, common breast cancers likely represent a continuum among common low-risk and rare high-risk genetic variants, which cumulatively contribute to the overall risk for each single person. For breast cancer, the rare high-risk genetic variants displaying a minor allele frequency (MAF) lower than 0.5% account for nearly 1–10% of disease incidence, allowing the identification of very high-risk individuals who could benefit from timely medical interventions. The remaining population does not show evidence of any familial disease (i.e., small families) or may have de novo genetic changes or a more complex genetic architecture. In fact, many high-(MAF > 5%) and low-frequency (MAF between 0.5% and 5%) genetic variants, which per se contribute small additive effects, may account for the nonfamilial risk of the disease. By combining together risk alleles, which are selected on the basis of the evidence derived from genome-wide association studies and weighted according to their effect size [138], polygenic risk scores (PRSs) have been established [137]. The PRS clinical validity is defined by the accuracy in the discrimination of the population under study into categories of different degrees of absolute risk, also in combination with clinical risk factors. In order to be used in clinical and preventive interventions, it is necessary that the PRS can be applied individually with a good balance between specificity (false-positive rate) and sensitivity (true-positive rate). The risk model’s discriminatory accuracy is assessed by calculating the area under the receiver operating characteristic curve (AUC), which represents the general probability that the predicted risk is higher for cases than for the control. Practically, the individual-level PRS values group the population based on risk percentiles (such as top 1%, top 10%), giving a different level of absolute probabilistic risk in each tier [139,140].

In breast and ovarian cancer, the first case–control study predicting BC [141] in women without any pathogenic variants in BRCA1/2 genes developed an SNP18 PRS with an OR of 1.55 (95% CI 1.29 to 1.87) and an AUC of 0.59, demonstrating the PRS feasibility. SNP77 was the next and well-approved BC PRS, based on around 33,000 BC cases and 33,000 European controls, which was able to stratify women with and without a family history of BC [139] with an OR of 1.52 (95% CI 1.45–1.59) and an AUC of 0.62.

**Figure 1:**
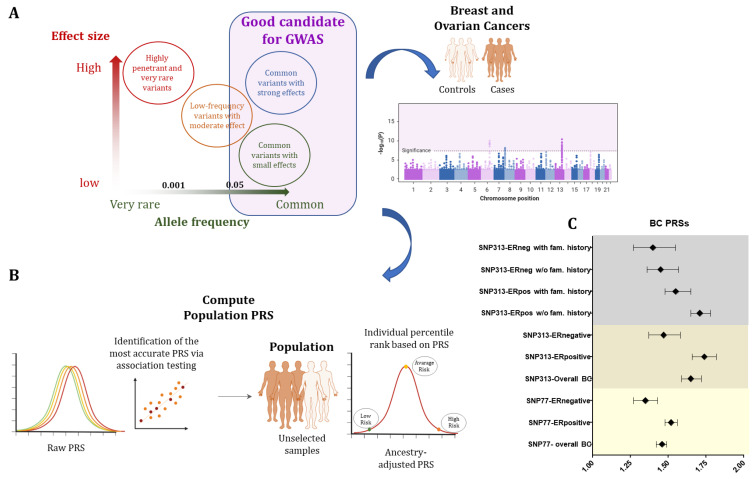
Polygenic risk scores (PRSs) in breast cancer risk. (**A**) Generation of PRSs by genome-wide association studies (GWASs) which identify SNV with various effect sizes on disease penetrance, to create numerous PRSs. (**B**) The various PRSs are then examined through different correlation testing, and the most accurate PRS is identified and then implemented in an independent-subject cohort. (**C**) Forest plot showing BC risk for specific breast cancer subtypes associated with 313- and 77-SNP PRSs from Mavaddat et al., 2019 [142].

The largest study of the Breast Cancer Association Consortium (about 11,000 cases vs. 18,000 controls) defined the most predictive SNP313 PRS for breast cancer risk development with an OR of 1.61 (95% CI 1.57–1.65) and an AUC of 0.63 in the European population [142]. Moreover, the BC lifetime risk in the top centile of the PRS was as high as 32.6% and women had 4.37- and 2.78-fold risks of developing both ER-positive and ER-negative diseases as compared to those in the intermediate quintile. Interestingly, both SNP77 and SNP313 are good and reliable predictors of ER-positive BC (subtype-specific PRSs), with more significant ORs than those for ER-negative tumors, possibly reflecting a higher statistical power of the cases with ER-positive tumors (Figure 1). Finally, when combined with the cancer family history, the PRS showed higher values in women without a family history (OR = 1.71, 95% CI 1.65–1.78) as compared to those with a positive family history (OR = 1.55 (95% CI 1.48–1.65) in the ER-positive subgroup [142].

It has also been demonstrated that 94-SNP and 18-SNP PRSs can predict absolute risks of developing BC and OC for *BRCA1/2* carriers [143]. The PRS for ER-negative women exhibited the highest correlation with BC risk in *BRCA1* patients with an HR of 1.27 (95% CI = ¼ 1.23 to 1.31); also, *BRCA2* carriers showed PRS for the risk of BC with HR = 1.22 (95% CI = 1.17 to 1.28), showing differences in absolute risks (more than 10% in each case) between the upper and lower deciles of the PRS curve [143].

Moreover, the following studies focused on implementing the breast and ovarian cancer SNP313 PRS for *BRCA1/2* carriers, to evaluate how its application may influence cancer risk guidance for women with pathogenic variants in these genes. Barnes et al. (2020) studied the largest cohort of *BRCA1/2*-positive women and further validated the associations in a prospective sample of carriers by applying population-based PRSs. *BRCA1* carriers showed the highest PRSs for ER-negative BC with a hazard ratio of 1.29 (95% CI 1.25–1.33), reflecting that those at the 5th and 95th percentiles of the PRSER distribution have predicted breast cancer risks to 80 years of age of 59% and 83%, respectively. Alongside this, *BRCA2* patients display the strongest correlation with the overall BC PRS and an HR of 1.31 (95% CI 1.27–1.36), which corresponds to risks of 57% and 81% for the *BRCA2* carriers in the 5th and 95th percentiles of the PRS. When evaluating ovarian cancer risk by the age of 80 years for *BRCA1* and *BRCA2* carriers, the PRSHGS (22 SNPs predicting high-grade serous OC) at the 5th and 95th percentiles showed risks of 30% and 59%, and 10% and 28%, respectively [144].

The risk stratification models have been further developed by integrating family history information, such as the age of disease onset and family breast cancer cases and other factors, such as endocrine and anthropomorphic factors, breast imaging density, lifestyle, and cases of previous benign proliferative disorders [145]. Several statistical models have included these factors, such as the Gail model, Breast and Ovarian Analysis of Disease Incidence and Carrier Estimation Algorithm (BOADICEA), Tyrer–Cuzick (TC), BRCAPRO, and Breast Cancer Surveillance Consortium (BCSC).

The first BOADICEA prediction model integrated the risk due to the rare loss-of-function variants in the “major genes” (*BRCA1* and 2, *PALB2*, *CHEK2* and *ATM*) with tumor pathology (receptors status), basic demographic element (e.g., year and country of birth and family ethnicity), and family history [146]. The authors also incorporated the SNP313 PRS, the mammographic density and questionnaire-based risk factors, to generate the first comprehensive model for the prediction of personal BC risk in unaffected women [146]. Thus, the integration of the effects of all known elements in a single model allows a more coherent method for risk stratification both in general individuals and in women with a family history of BC, with the major limitation represented by the reference population bias, due to the assessment of an only-European population in the computation of the PRS.

More recently, PRS has improved also via artificial intelligence technologies and a deep neural network (DNN), and other computational models (logistic regression, decision tree, random forest, AdaBoots, gradient boosting, support vector machine (SVM), and Gaussian naïve Bayes) have been tested to estimate breast cancer PRS starting from 5273 SNPs as input [147]. For this study, a breast cancer GWAS dataset containing 49,111 individuals of the DRIVE (Discovery, Biology, and Risk of Inherited Variants in Breast Cancer) project obtained from the NIH dbGaP database (accession number: phs001265.v1.p1.) was employed [148]. The top-100 relevant SNPs identified the individuals with the highest PRS (>0.67 cutoff) with a 90% precision and, among those SNPs, 8 belonged to genes with a functional role in BC pathogenesis [147]. AI tools based on PRS have also been used to reduce screening costs, as emerged in some recent studies [149,150,151]. Mital et al.’s [149] economic evaluation using a hybrid decision tree/microsimulation model for the comparison of costs and effectiveness found that AI-based risk prediction and the subsequent lack of screening for women with low susceptibility was the most efficient and budget-conscious approach. Thus, AI was more accurate in identifying high-risk women than family history when used in conjunction with PRS, achieving fewer false-positive diagnoses from not screening low-risk women.

**Figure 2:**
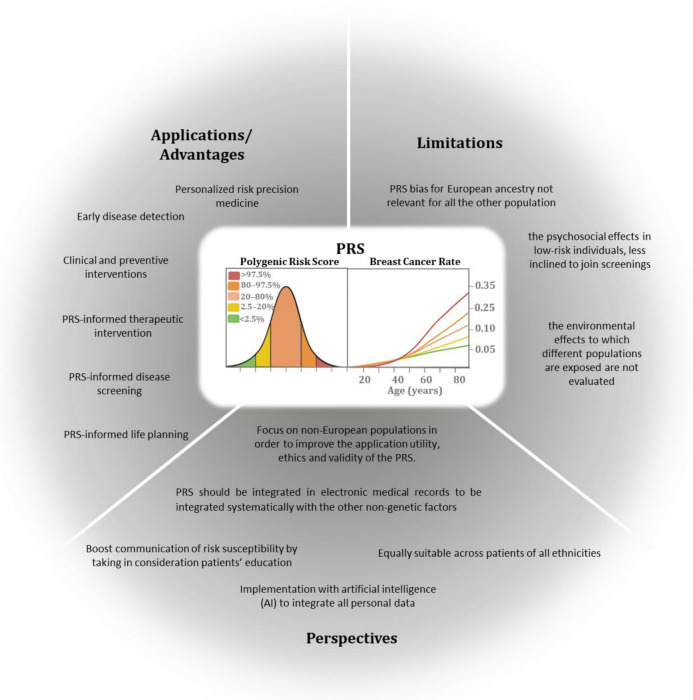
Benefits, limitations, and future perspectives of PRS in breast cancer management.

In conclusion, BC PRS is an increasingly successful attempt to establish a personalized medicine for risk prediction (Figure 2) in unaffected subjects as compared to classic risk factors, although further studies are still needed to understand the potential contribution of other novel technologies (e.g., AI) and to improve the predictive power beyond the ancestry-dependent genetic background.

## 8. Homologous Recombination Deficiency (HRD)

**Figure 3:**
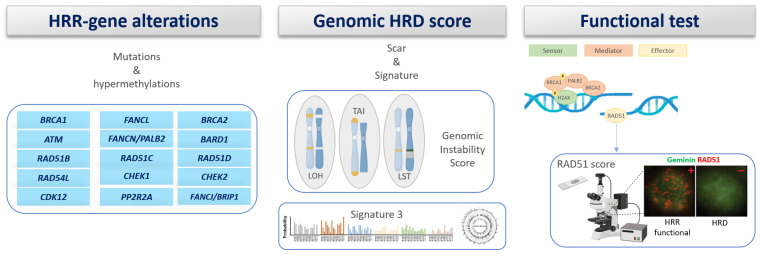
Homologous recombination repair deficiency (HRD) detection is based on various methods based on germline or somatic analysis.

The homologous recombination deficiency (HRD), which is intrinsic to the HBOC-associated cancers, has been exploited both in therapy (Table 3), which is based on the suppression of alternative repair pathways (PARP inhibition), and in diagnostics (Figure 3), by tracing the genomic lesions attributable to the diminished activity in the Homologous Recombination Repair pathway, called genomic scars. 

### 8.1. Gene Scar/Signature

Cancer cells and cells with *BRCA1/2* variants are characterized by genomic instability: they exhibit copy number modifications and numerous somatic changes in the genome, including single-nucleotide polymorphisms and structural aberrancies (structural variants, SVs). The evaluation of these genomic characteristics allows the identification of tumors with a history of HRD, regardless of the underlying etiology.

Genomic HRD tests have been developed using SNP microarrays, measuring somatic CNVs. In 2012, three papers reported SNP-based CNV tests that calculated BRCA status by quantifying large-scale transitions (LSTs), loss of heterozygosity (LOH), and the number of sub-chromosomal regions with an allelic imbalance up to the telomere (TAI/telomeric allelic imbalance) [152].

Two commercial tests related to genomic scars have been approved by the FDA and used to identify tumors with HRD: (1) the “myChoice HRD” kit by Myriad, which inquires the LOH, the TAI, and the LST throughout the genome [153], and (2) the “FoundationFocus™ CDx BRCA LOH”, which is conceived to identify the occurrence of variants in *BRCA1/2* and the amount of the genome impacted by LOH in tumor-derived DNA of patients with OC [154]. Finally, the non-commercial test HRDetect [155] includes additional mutational profiles and characteristics derived by each mutational mechanism on the tumor genome: for instance, HRD has been correlated with the “signature 3” characterized by Alexandrov et al. [156,157].

### 8.2. Functional Assays of HRD

A key constraint of the gene scar tests is the lack of evaluation of the processes of tumor progression, such as a recovery of the HRR process in response to treatment-driven pressure. Instead, it might be advantageous to integrate functional biomarkers developed on assays capable of estimating the functionality of a repair pathway in a dynamic way. A crucial step of HRR is mediated by the RAD51 protein, a nucleoprotein filament, which is accomplished to execute the strand exchange step of HRR [158]. Despite some limitations yet to be solved, several approaches, both *in vivo* and *in vitro*, underpinned the highly sensitive and specific predictive value of the absence of nuclear RAD51 foci in response to PARPi therapy (Table 4) [159,160,161,162]. One such drawback is that the RAD51 test might fail to identify *ATM*-variant-associated tumors that can benefit from PARPi according to their specific mechanisms of sensitivity [163,164,165,166].

### 8.3. RAD51 Assay as Functional Biomarker of HRD in Early BC

A retrospective study of the accuracy of the RAD51 assay, which was based on 133 tumors from TNBC patients enrolled in the GeparSixto trial, has shown that in *BRCA1*-mutated tumors, the nuclear foci level (BRCA1 score) does not occur in 43% of tumors, which were also with low RAD51 levels. The RAD51 prognostic marker detected 93% (76–99%, 95% CI) of t*BRCA1/2*-mutated cancer and 45% (34–56%, 95% CI) of the non-t*BRCA1/2* mutants with active HRD. RAD51 detected 86% of cancers with HRD and 90% with HRR proficiency (HRP). Overall, RAD51 and genomic HRD were concordant in 87% (79–93%), demonstrating the feasibility of the RAD51 assay in its application on FFPE samples derived from untreated TNBC and its high degree of congruence with t*BRCA1/2* mutations and HRD results [167].

When used for monitoring the response to chemotherapy, the RAD51 assay can significantly distinguish cancers responsive to carboplatin (*p* = 0.02): the benefit of its administration was maintained also in the multivariate analysis after adjustment for predefined clinical–pathological parameters (OR = 7.52, 95% CI 2.21–25.61, *p* = 0.001). Moreover, in terms of DFS (disease-free survival), a similar response was observed in RAD51-high and RAD51-low groups, whereas in OS, no evident correlation was reached [167].

The RAD51 assay was also performed on samples from early TNBC patients enrolled in the PETREMAC trial and treated with PARPi Olaparib in a neoadjuvant setting. Functional HRD, with low RAD51 scores, is associated with HR mutations/*BRCA1* methylation profiles, as well as Olaparib response [168].

**Table 3:** Efficacy of PARPi according to HRD status in ovarian cancer.

Clinical Trial	Drug	Setting	Study Population	HRD Role
ARIEL-2	Rucaparib	Monotherapy	Relapsed, platinum-sensitive ovarian cancer	Higher performance in g*BRCA1/2*-variant-associated and/or LOH-high compared to LOH-low tumors. Not effective in manifesting a difference between LOH-high and LOH-low tumors
ARIEL-3	Rucaparib	Maintenance therapy	Platinum-sensitive ovarian cancer	Efficacy regardless of LOH status.
NOVA-trial	Niraparib	Maintenance therapy	Platinum-sensitive ovarian cancer	Efficacy regardless of HRD status.

**Table 4:** Efficacy of PARPi according to HRD status in breast cancer.

Clinical Trial	Drug	Setting	Study Population	HRD Role
PrECOG 0105 Cisplatin-1 trial Cisplatin-2 trial	Platinum salts	Neoadjuvant setting	Untreated patients	HRD-positive patients had higher complete pathologic response
Gepar-Sixto trial	Carboplatin	Neoadjuvant setting	Untreated patients	HRD-positive patients have a better prognosis by comparing with HRD-negative ones. No robust evidence can be reached about the predictive role of HRD regarding carboplatin
TBCRC009 trial	Platinum salts	Advanced setting	First- or second-line treatment	Higher HRD scores were reported in responding patients, independent of *BRCA1/2* variant status.
TNT trial	Carboplatinum	Advanced setting	First-line treatment	ORR is not associated with HRD levels in the primary tumors.

## 9. Conclusions and Perspectives

Once established, the contribution of high-penetrance mendelian genes to the pathogenesis of HBOC, however, contributes only to a small share of the whole breast cancer burden, and the paradigm shifts to the study of the genome in search of variants to be used for prognosis or therapy. Still, there is a long way to go, and even genes which have been tested for diagnostic purposes in millions of women worldwide are still frequently (~5%) burdened by the presence of variants of uncertain significance, which deprive the result of the test of its clinical significance and may result in inappropriate clinical management [169,170]. Crucially, genetic findings related to the overall *BRCA1/2* variants were conducted mainly on Caucasian individuals. This fundamental fact is primarily due to the significant absence of knowledge in the genetic variation for all other ethnic populations, highlighting the issue of understanding the full spectrum of pathogenicity of the current variant information related to BRCA genes, which may not be applicable to other ethnicities. In this perspective, future studies might decipher the proper genetic landscape underneath BRCA genes only after inclusion of a broader ethnicity in the gene analysis with the final aim of giving more focused clinical interventions. Moreover, the HBOC has contributed to reduce the borders between mendelian and multifactorial disorder categories, also showing the deep interaction between the two factors: for example, the probability of BC occurrence by age 75 spans from 12.7% to 75.7% among *BRCA1* and *BRCA2* pathogenic variant carriers, while the risk of non-carriers oscillates between 3.3% and 29.6%, showing a high overlap between carriers and non-carriers of high-risk variants, which can be explained only in terms of the polygenic risk of other low-penetrance variants. HBOC has always been ahead of all the other hereditary cancer predisposition syndromes in terms of knowledge, protocols, and specific therapeutic options available: replicating the efforts and successes achieved for HBOC should inspire future research for other cancer predisposition syndromes and for their sporadic counterparts.

## Figures and Tables

**Table 1:** Genes associated with breast and ovarian cancers. * lifetime risk.

Gene	BC Risk *	OC Risk *	Other Cancer Risk
*BRCA1*	60–66%	41–58%	Pancreatic cancer
*BRCA2*	55–61%	15–16%	Pancreatic and Prostate cancer
*ATM*	20–40%	2–3%	Pancreatic, Prostate cancer
*BARD1*	20–40%	Not Assessed	Insufficient Evidence
*BRIP1*	Not Assessed	5–15%	Insufficient Evidence
*CDH1*	41–60%	Not Assessed	Hereditary diffuse gastric cancer
*CHEK2*	20–40%	Not Assessed	Colorectal, kidney, thyroid cancer
*PALB2*	41–60%	3–5%	Pancreatic cancer
*PTEN*	40–60%	Not Assessed	Colorectal, renal, thyroid cancer
*RAD51C*	20–40%	10–15%	Insufficient Evidence
*RAD51D*	20–40%	10–20%	Insufficient Evidence
*TP53*	60%	Not Assessed	Brain tumors, sarcoma, acute leukemia, adrenocortical tumors
